# Tardive peritonitis after the endoscopic ultrasound‐guided hepaticogastorostomy: A case report

**DOI:** 10.1002/deo2.77

**Published:** 2021-11-24

**Authors:** Yukitaka Yasuhara, Nana Shimamoto, Shintaro Tsukinaga, Masayuki Kato, Kazuki Sumiyama

**Affiliations:** ^1^ Faculty of Medicine the Jikei University School of Medicine Tokyo Japan; ^2^ Department of Endoscopy the Jikei University School of Medicine Tokyo Japan

**Keywords:** endoscopic ultrasound‐guided biliary drainage, endoscopic ultrasound‐guided hepaticogastrostomy, gastric outlet obstruction, pancreatic cancer, peritonitis

## Abstract

Endoscopic ultrasound‐guided biliary drainage (EUS‐BD) has become popular as a new drainage technique for malignant biliary strictures. Although EUS‐BD has been reported to show high technical and clinical success rates, the rate of adverse events is 15%. In particular, peritonitis, which is generally caused by bile leakage from the aspiration side during the procedure and occurs within a few days after EUS‐BD, needs to be considered as it can be fatal. In the present case, a jaundiced patient presented with unresectable pancreatic adenocarcinoma. Due to duodenal invasion, we performed EUS‐guided hepaticogastrostomy for biliary drainage. After the procedure, jaundice improved, and abdominal computed tomography (CT) showed only a small amount of air in the intrahepatic bile duct. However, 7 days after the procedure, the patient developed fever, and clinical findings indicated peritonitis. Abdominal CT showed food in the stomach accompanied by the appearance of perihepatic free air, with increased air in the intrahepatic bile duct. The duodenal stent insertion settled the peritonitis and improved the perihepatic free air and the air in the intrahepatic bile duct through the discharge of food from the stomach. To date, no case of tardive peritonitis associated with air leakage after EUS‐BD has been reported. We noted that even if there was no evidence of bile leakage after EUS‐BD, the possibility of tardive peritonitis due to gradual air leakage from the stent implantation side of the stomach should be considered, and careful follow‐up is needed.

## INTRODUCTION

Endoscopic ultrasound‐guided biliary drainage (EUS‐BD) is gaining popularity as a salvage modality for malignant biliary strictures for which transpupillary drainage fails.^1^, ^2^, ^3^ Although the technical and clinical success rates exceed 90%, serious adverse events, such as perforation, sepsis, and peritonitis, have been reported.^1^, ^4^ In particular, peritonitis requires intensive care because it can develop into sepsis through bacterial translocation from the abdominal cavity to the blood vessels; some fatal cases have been reported.^1^, ^4^, ^5^ We encountered an atypical case of peritonitis, which differed from previous reports. We believe that this report may assist in the establishment of safer procedures for EUS‐BD and the development of new devices in the future.

## CASE REPORT

An 86‐year‐old woman complained of jaundice and itchy skin. In late April 2021, the patient's skin began to itch, and in early May, yellowing of the skin was noted. Therefore, she visited a general physician. Abdominal ultrasonography revealed dilated intrahepatic bile ducts, and the patient was referred to our hospital. Her medical history was significant only for type 2 diabetes, social alcohol consumption, and a family history of pancreatic cancer. Her height was 155 cm; weight, 52 kg; body mass index, 18; body temperature, 37.0°C; blood pressure, 140/75 mmHg; and pulse, 82 beats per minute. Physical examination revealed yellow discoloration of the skin and conjunctiva icterus.

The anomalies in the blood indicated the malignant biliary stricture as follows: total bilirubin, 8.9 mg/dl; direct bilirubin, 7.2 mg/dl; aspartate aminotransferase, 352 IU/L; alanine aminotransferase, 478 IU/L; lactate dehydrogenase, 345 IU/L; cholinesterase, 188 U/l; γ‐glutamyl transpeptidase, 617 IU/L; alkaline phosphatase, 825 IU/L; carbohydrate antigen, 19‐9 1246 U/ml; DUPAN‐2, 4900 U/ml; and Span‐1, 550 U/ml.

**FIGURE 1:**
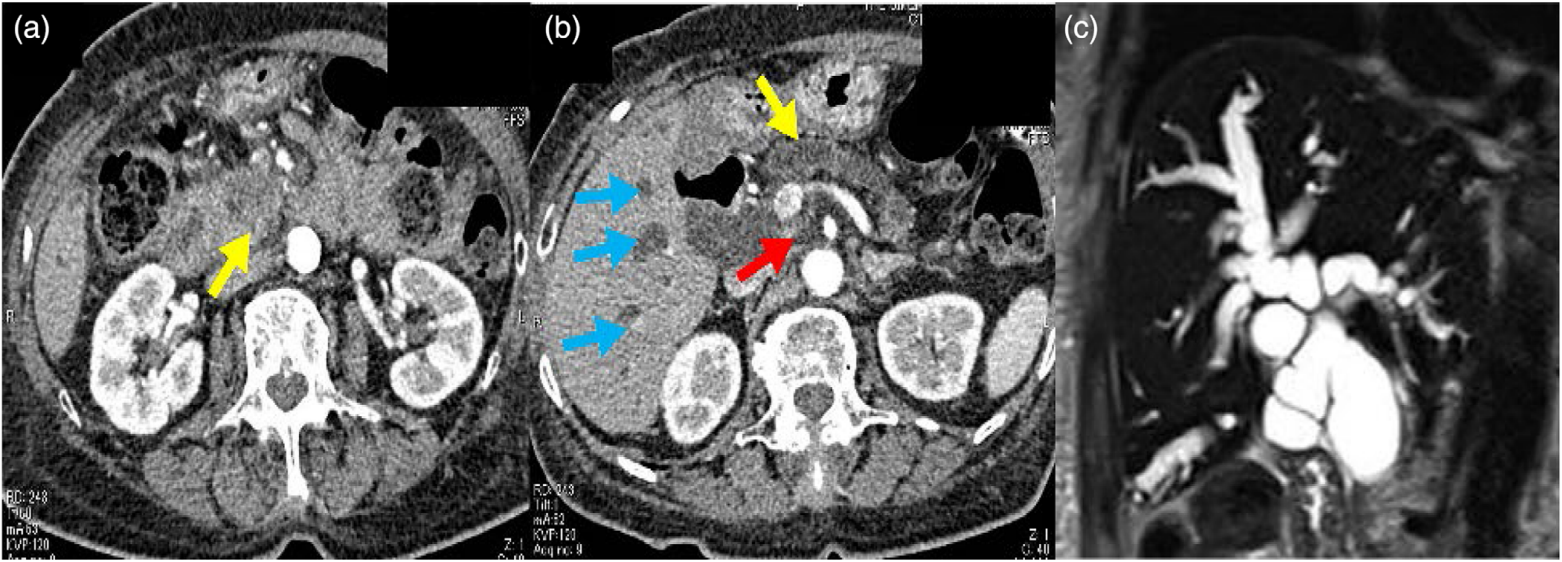
Abdominal contrast computed tomography and magnetic resonance cholangiopancreatography images. (a) Contrast‐enhanced computed tomography arterial phase. A 40‐mm large low‐density area is observed at the head of the pancreas (arrow). (b) Contrast‐enhanced computed tomography. Infiltration of the celiac and superior mesenteric arteries is observed (yellow arrow). The main pancreatic duct is dilated (red arrow). Multiple hepatic metastases are revealed (blue arrow); however, there are no significant lymph node metastases. (c) Magnetic resonance cholangiopancreatography showing dilatation of the bile duct and main pancreatic duct

**FIGURE 2:**
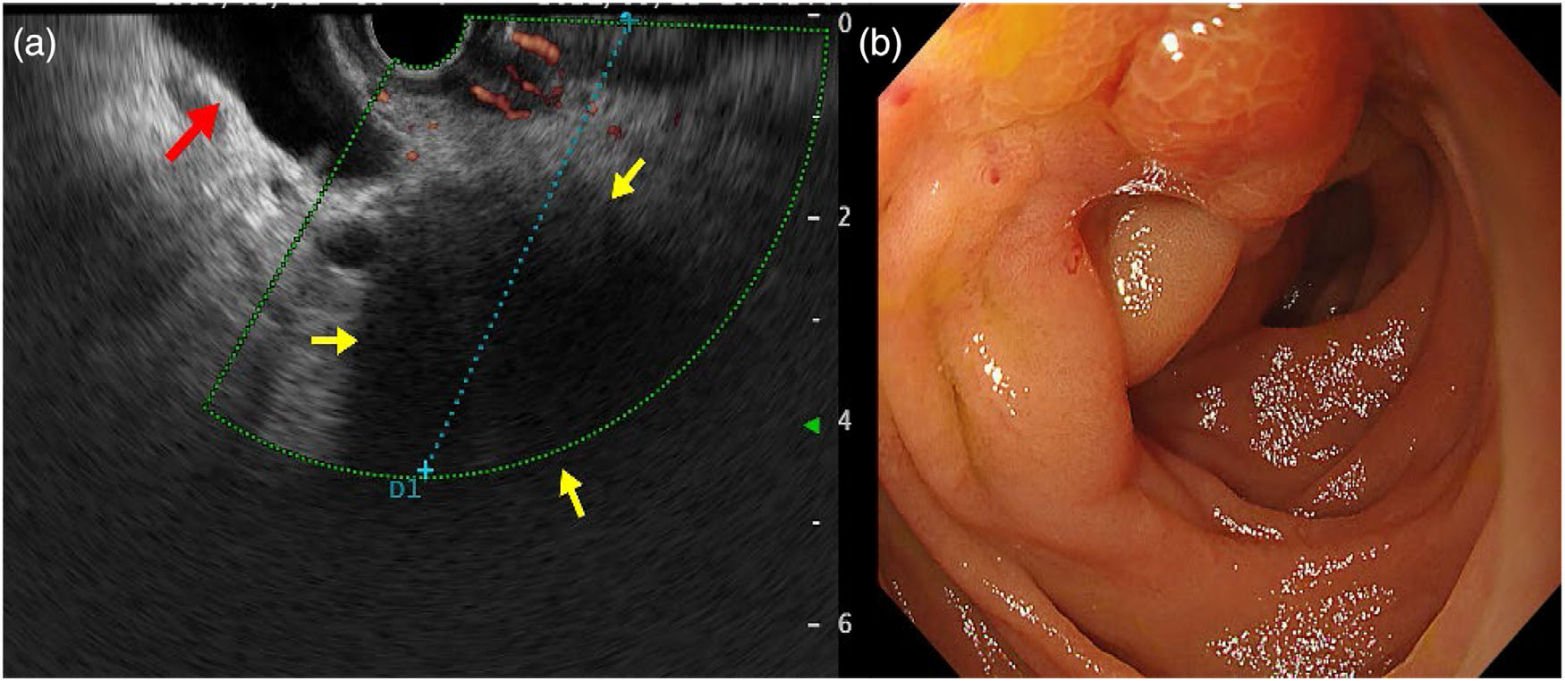
Endoscopic ultrasonography and upper endoscopy images. (a) Endoscopic ultrasonography images. A hypoechoic mass of 40 mm in size is seen in the pancreatic head (yellow arrow), and the common bile duct is disrupted at that site and dilated upstream (red arrow). (b) Upper endoscopy shows stenosis with mucosal irregularity on the anal side of the Vater papilla. A biopsy was performed at the stenotic site, and the pathological diagnosis was adenocarcinoma

Abdominal contrast‐enhanced computed tomography (CT), magnetic resonance cholangiopancreatography (MRCP), endoscopic ultrasonography, and upper endoscopy were performed. Abdominal contrast‐enhanced CT showed a 40‐mm low‐density area in the pancreatic head and multiple metastases in the liver (Figure 1a,b). MRCP showed dilatation of the bile and the main pancreatic ducts (Figure 1c). Endoscopic ultrasonography showed a 40‐mm hypoechoic area and dilated bile ducts (Figure 2a). An esophagogastroduodenoscopy revealed stenosis of the duodenum (Figure 2b). A biopsy of the duodenal stenosis showed adenocarcinoma. Thus, the patient was diagnosed with pancreatic head cancer (stage IV; T4 N0 M1). We tried endoscopic retrograde cholangiopancreatography (ERCP) before performing EUS‐BD but the ERCP scope (JF260 duodenoscope; Olympus Corporation, Tokyo, Japan) was not able to pass through the constricted superior duodenal angle caused by the duodenal invasion of the tumor.

**FIGURE 3:**
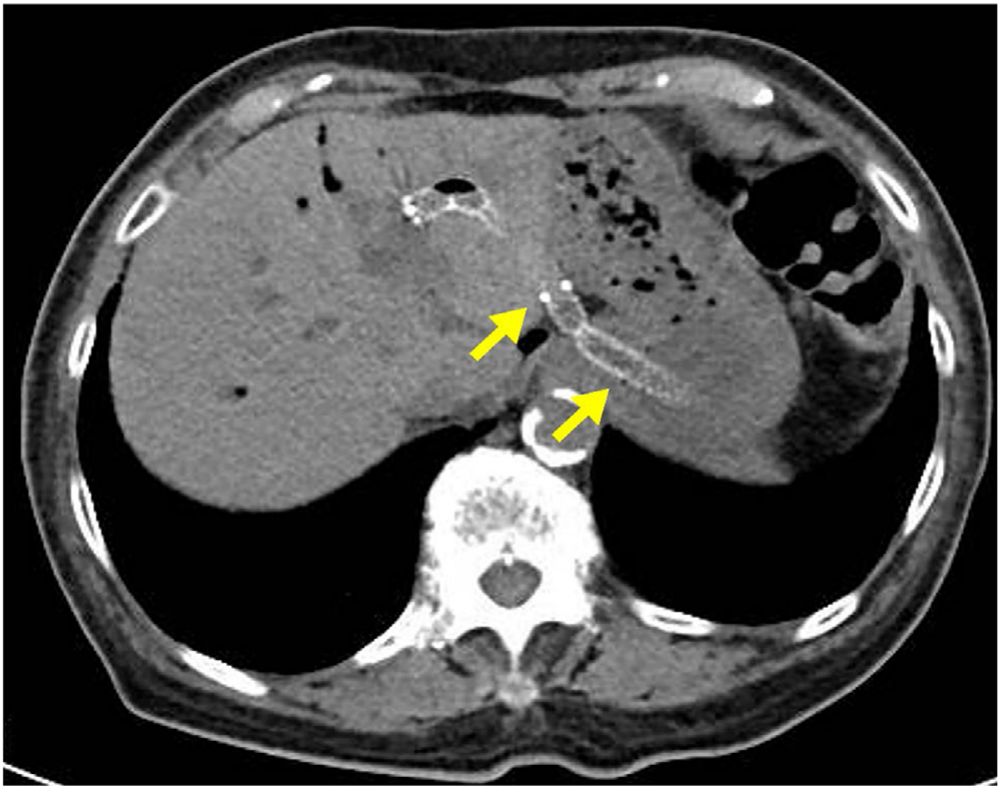
Abdominal computed tomography shows the resolution of the intrahepatic hepatic duct dilation and the placed stent (arrow)

On the 8th day after hospital admission, EUS‐guided hepaticogastrostomy (EUS‐HGS) was performed to treat the cholangitis associated with obstructive jaundice. Under EUS guidance, the left B3 duct was punctured with a 19‐gauge needle (EZ shot 3 Plus, 19‐gauge; Olympus), and a 0.025‐inch guidewire (VisiGlide2, 0.025 inches; Olympus) was inserted into the bile duct. The puncture tract was dilated with a diathermic dilator (Cysto‐Gastro‐Set, 6Fr; Century Medical Inc, Tokyo, Japan). Thereafter, a fully covered self‐expanding metallic stent (HANAROSTENT, 6 mm 120 mm; Boston Scientific, Massachusetts, USA) was deployed. On the ninth day after hospital admission, jaundice was relieved, and liver function improved. Abdominal CT showed improvement in intrahepatic bile duct dilation (Figure 3).

**FIGURE 4:**
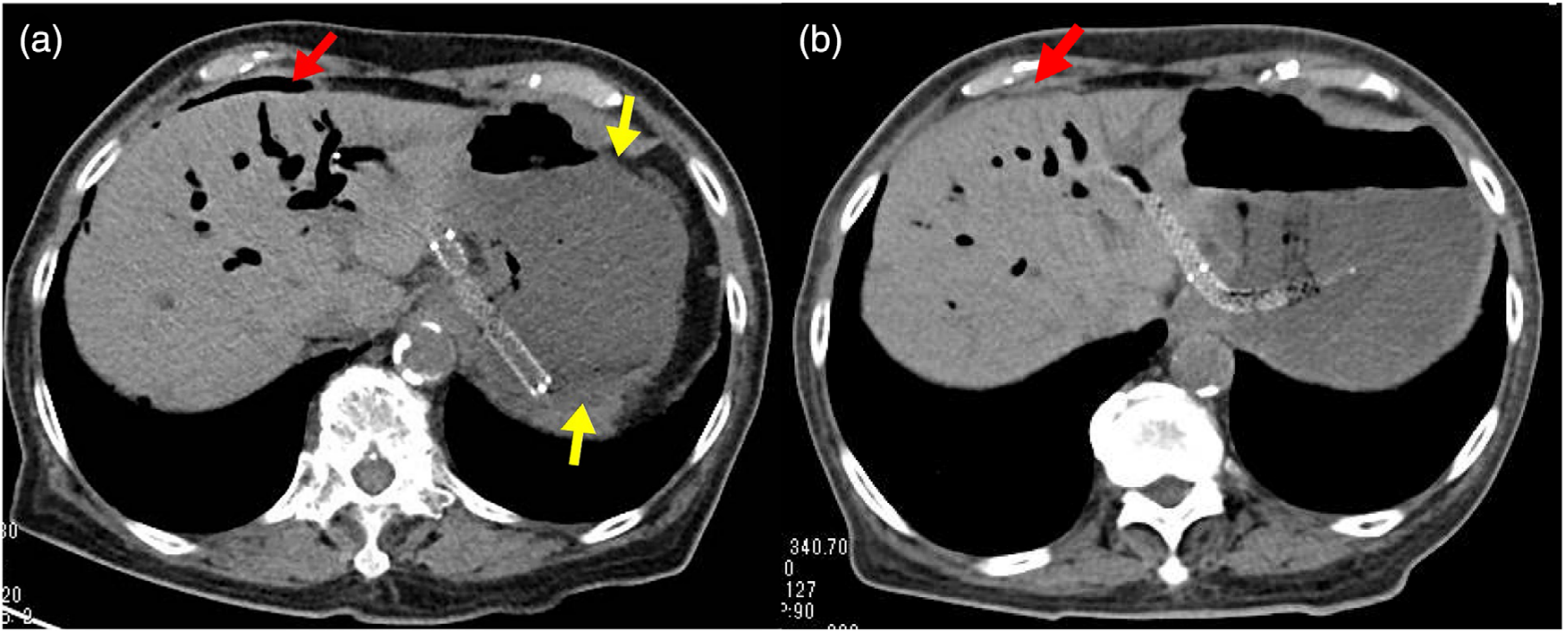
Comparison of the volume of free air between before and after the duodenal stent insertion by abdominal computed tomography. (a) When the patient complained of fever and abdominal pain, Abdominal computed tomography shows free air around the liver (red arrow) with food stored in the stomach (yellow arrow). (b) After the duodenal stent insertion, free air around the liver decreased (red arrow) compared with that before the procedure

On the 11th day, a liquid diet was started, and on the 15th day after hospital admission, the patient complained of fever and abdominal pain. Examination revealed mild tenderness, abdominal guarding throughout the abdomen, and peritoneal irritation with ambulation. As for the blood sampling data when the patient showed symptoms, though inflammatory response increased (white blood cell count, 12300 /μl and C‐reactive protein, 4.2 mg/dl), bilirubin and hepatobiliary enzyme kept decreasing after EUS‐BD and did not elevate (total bilirubin, 5.3 mg/dl; direct bilirubin, 4.8 mg/dl; aspartate aminotransferase, 36 IU/L; alanine aminotransferase, 90 IU/L; γ‐glutamyl transpeptidase, 231 IU/L; and alkaline phosphatase, 321 IU/L). Abdominal CT showed that the food was stored in the dilated stomach, and free air around the liver had increased with dilated bile duct at B3 (Figure 4). Based on the clinical and imaging findings with the transition of the blood sampling data, though there was room for obstructive cholangitis due to self‐expanding metallic stent (SEMS) implantation, peritonitis was suspected. Tazobactam/piperacillin started on the 16th day made the inflammatory response turn to decline but the patient still complained of mild abdominal pain. On the 22nd day after hospital admission, a duodenal stent was placed to alleviate the duodenal obstruction. After stent placement, both gastrointestinal obstruction and peritonitis were relieved with the reduction of free air around the liver (Figure 4), and peritonitis did not recur even after eating a solid diet. The patient was discharged on the 34th day after hospital admission.

## DISCUSSION

Peritonitis is an adverse event of EUS‐BD, which occurs at an incidence rate of 1% and fatal cases have been reported; therefore, it is a cautionary complication. The cause is reported to be bile leakage associated with the EUS‐BD procedure.^6^ Bile leakage exposes bacteria in the infected bile to the abdominal cavity, and irritation of the mesenteries due to the toxicity of bile acids induces bacterial translocation, leading to peritonitis.^7^

In the present case, there was gastric outlet obstruction (GOO) due to duodenal invasion of pancreatic cancer. Although GOO was resolved by the duodenal stent insertion, in this case, the risk of HGS stent deviation by the duodenal stent insertion should be taken into consideration. GOO accelerated the intragastric pressure and imaging findings at the onset of peritonitis showed food stored in the stomach and increased free air in the abdominal cavity, but no bile leakage. Although there remained the possibility of a small amount of bile leak that is not evident on CT, it was determined that the cause of the peritonitis was not bile leakage, but rather air leakage accompanied by the visceral contents into the peritoneal cavity. These are only predictions because the blood culture at the time of peritonitis onset was negative but considering that the positivity rate of blood culture in biliary tract infections is only approximately 5%,^8^ the estimation from the imaging findings is reasonable. Moreover, the previous report accessing the cause of peritonitis induced by the air leakage, which showed that air leakage of postoperative status, was the third common cause of the peritonitis, and the most common anatomical site of air leakage was gastroduodenal legion might support our opinion.^9^

A fully covered SEMS (FCSEMS) is often used for EUS‐HGS to ensure patency and reduce bile leakage from the fistula between the stomach and hepatic duct.^2^, ^10^ In recent years, SEMS with only the distal end uncovered to avoid obstruction of the bile duct and stents with a small diameter to match the bile duct diameter have been introduced. In this case, we used a 6‐mm stent. Although there are no reports showing a difference in the type or frequency of adverse events depending on the diameter of the FCSEMS, it cannot be denied that the narrow diameter may have contributed to air leakage through the gap between the stomach and the stent with other possibilities such as a broken cover section.

We encountered a case of peritonitis that developed as an incidental complication of EUS‐BD 7 days after treatment. The leak was thought to be caused by air leakage accompanied by the visceral contents into the peritoneal cavity due to increased intragastric pressure induced by duodenal obstruction after the start of eating, rather than bile leakage caused by the procedure itself. Even if bile leakage is mild on imaging findings after EUS‐BD, late onset of peritonitis may occur. It is important to carefully consider the selection of stents, treatment of coexisting duodenal obstruction and formulate a strategy.

## ETHICS STATEMENT

This case report does not contain any analysis with human or animal subjects performed by any of the authors. Informed consent about writing the case report was obtained from the patient.

## CONFLICT OF INTEREST

All authors had full access to the data and had final responsibility for the decision to submit for publication. No benefits in any form have been received or will be received from a commercial party related directly or indirectly to the subject of this article. Kazuki Sumiyama is a Deputy Editor‐in‐Chief of DEN Open.

## FUNDING INFORMATION

We did not receive any funding support for this report.
